# Rac1-Regulated Endothelial Radiation Response Stimulates Extravasation and Metastasis That Can Be Blocked by HMG-CoA Reductase Inhibitors

**DOI:** 10.1371/journal.pone.0026413

**Published:** 2011-10-19

**Authors:** Melanie Hamalukic, Johannes Huelsenbeck, Arno Schad, Stefan Wirtz, Bernd Kaina, Gerhard Fritz

**Affiliations:** 1 Institute of Toxicology, University Medical Center of the Johannes Gutenberg University Mainz, Mainz, Germany; 2 Institute of Pathology, University Medical Center of the Johannes Gutenberg University Mainz, Mainz, Germany; 3 Institute of Molecular Medicine, University Medical Center of the Johannes Gutenberg University Mainz, Mainz, Germany; 4 Institute of Toxicology, Heinrich Heine University Düsseldorf, Düsseldorf, Germany; The Beatson Institute for Cancer Research, United Kingdom

## Abstract

Radiotherapy (RT) plays a key role in cancer treatment. Although the benefit of ionizing radiation (IR) is well established, some findings raise the possibility that irradiation of the primary tumor not only triggers a killing response but also increases the metastatic potential of surviving tumor cells. Here we addressed the question of whether irradiation of normal cells outside of the primary tumor augments metastasis by stimulating the extravasation of circulating tumor cells. We show that IR exposure of human endothelial cells (EC), tumor cells (TC) or both increases TC-EC adhesion *in vitro.* IR-stimulated TC-EC adhesion was blocked by the HMG-CoA reductase inhibitor lovastatin. Glycyrrhizic acid from liquorice root, which acts as a Sialyl-Lewis X mimetic drug, and the Rac1 inhibitor NSC23766 also reduced TC-EC adhesion. To examine the *in vivo* relevance of these findings, tumorigenic cells were injected into the tail vein of immunodeficient mice followed by total body irradiation (TBI). The data obtained show that TBI dramatically enhances tumor cell extravasation and lung metastasis. This pro-metastatic radiation effect was blocked by pre-treating mice with lovastatin, glycyrrhizic acid or NSC23766. TBI of mice prior to tumor cell transplantation also stimulated metastasis, which was again blocked by lovastatin. The data point to a pro-metastatic trans-effect of RT, which likely rests on the endothelial radiation response promoting the extravasation of circulating tumor cells. Administration of the widely used lipid-lowering drug lovastatin prior to irradiation counteracts this process, likely by suppressing Rac1-regulated E-selectin expression following irradiation. The data support the concern that radiation exposure might increase the extravasation of circulating tumor cells and recommend co-administration of lipid-lowering drugs to avoid this adverse effect of ionizing radiation.

## Introduction

Ionizing radiation (IR) is frequently used in cancer therapy to achieve local tumor control. Despite of the enormous merit of radiotherapy in the treatment of malignant diseases, it is well known to cause not only tumor cell death but also normal tissue damage that results in inflammation and fibrosis [1], [2]. Another side effect of IR rests on its ability to change the geno- and phenotype of tumor cells that have survived radiation exposure, leading to increased malignancy. Thus, *in vitro* studies demonstrated a gain of motility, adhesion and invasiveness of tumor cells upon irradiation, which are based on complex changes in gene expression, among others the up-regulation of matrix metalloproteinases (MMP) [3], [4], [5], [6], [7]. A variety of preclinical *in vivo* studies suggest that IR-induced stress responses of surviving tumor cells might promote their invasive potency [8], [9], [10], [11]. Moreover, pro-angiogenic effects of ionizing radiation have been reported [12], although a report exists claiming the opposite to be the case [13]. Occasional observations in patients are assisting the concern of adverse pro-metastatic radiation effects [14], [15], [16]. Therefore, failure to achieve local tumor control is suspected to promote the dissemination of single tumor cells from the primary tumor and their subsequent invasion into the normal tissue. The clinical importance of the pro-metastatic radiation effect is controversially discussed [17], [18].

Apart from tumor cells, the normal tissue is also exposed to radiation, e.g. during prophylactic cranial irradiation (PCI) or when large parts of the body are irradiated, e.g. for the treatment of Hodgkins disease or for palliative purposes. Low dose exposure of normal cells is even unavoidable in the course of local tumor irradiation. It is conceivable, yet poorly investigated to date, that irradiation of the normal tissue might also stimulate metastasis. This could occur for example by enhanced extravasation of tumor cells that are circulating in the blood at the time of radiotherapy. This possible side effect of radiotherapy, which has not been addressed yet, might be evoked by upregulation of endothelial adhesion factors required for the binding and extravasation of malignant cells. In this process, endothelial selectin (E-selectin), which is upregulated by IR both on the level of the mRNA and protein [19], [20], is of particular importance [7], [21] as it mediates the interaction of the endothelium with tumor cells that specifically express sialylated carbohydrate structures operating as E-selectin ligands [21], [22], [23], [24], [25].

A key response of both normal and tumor cells to IR is the activation of the transcription factor nuclear factor-kappaB (NF-κB) [26], [27], which regulates cell survival [28], [29] and inflammation [30], [31]. Bearing in mind that the expression of a variety of cell adhesion molecules [32], [33], including E-selectin [34], [35], depends on the transcription factor NF-κB, pharmacological interference with IR-induced activation of NF-κB is a rational strategy for modulating non-beneficial radiation responses of normal tissue. Previously, we showed that HMG-CoA reductase inhibitors (statins), which are widely used in the clinic for lipid lowering reasons, are able to attenuate the endothelial activation of NF-κB provoked by inflammatory cytokines and IR [7], [36]. This statin effect rests on the inhibition of the activity of Ras-homologous (Rho) GTPases [37] that are key regulators of NF-κB [38].

Here we addressed the questions of whether (i) IR can enhance the binding of tumor cells to the endothelium thus assisting metastasis and (ii) statins are able to mitigate IR-stimulated metastatic processes. We show that IR stimulates tumor cell-endothelial cell adhesion *in vitro,* which is based on the upregulation of pro-adhesive factors in both tumor and endothelial cells. We extended these studies to an *in vivo* model of metastasis. The data revealed that whole body irradiation of mice that had been transplanted with tumorigenic cells by intravenous injection leads to a significant increase in lung metastases as compared to non-irradiated animals. We further provide evidence that the IR-stimulated extravasation and metastasis can be antagonized by pre-administration of the lipid lowering drug lovastatin.

## Results and Discussion

### Pro-adhesive radiation responses of human tumor and endothelial cells are affected by lovastatin *in vitro*

**Figure 1:**
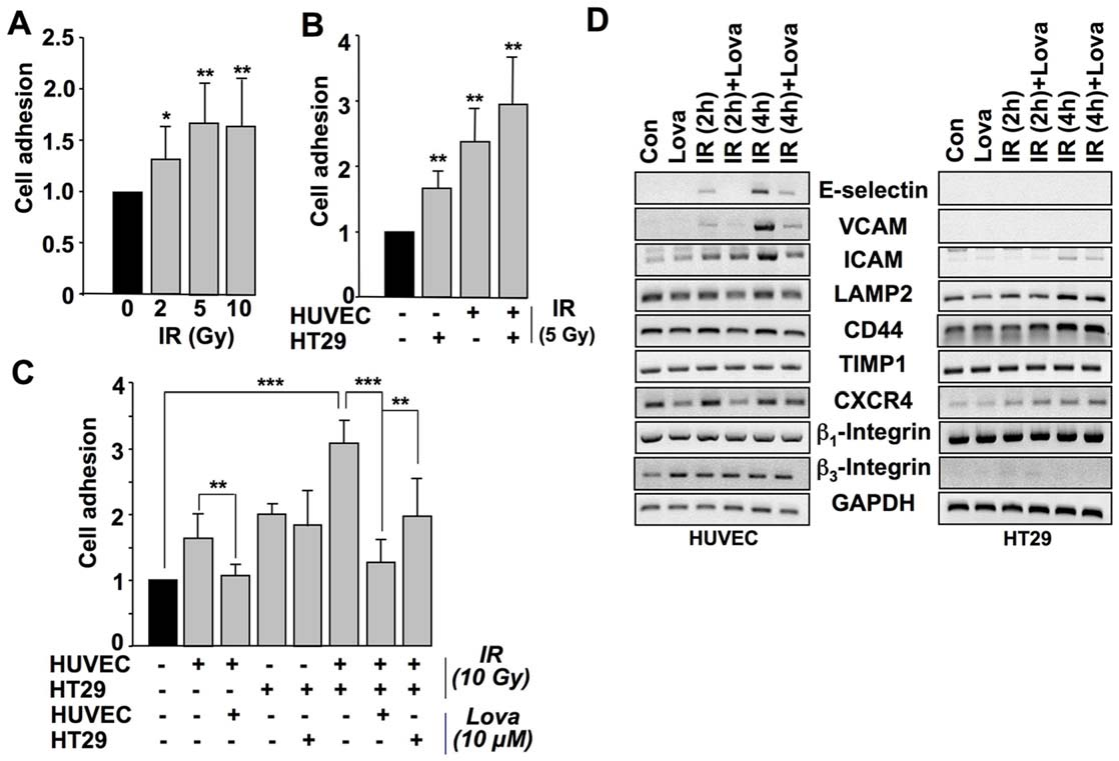
Irradiation of both endothelial and tumor cells stimulates TC-EC adhesion that is attenuated by lovastatin. A: Human colon carcinoma cells (HT29) were irradiated with 2–10 Gy. 4 h later, adhesion to primary human endothelial cells (HUVEC) was analyzed as described in Methods. Relative cell adhesion in untreated control was set to 1.0. *p≤0.05, **p≤0.01 (n = 15). B: Human colon carcinoma cells (HT29) or endothelial cells (HUVEC) were irradiated with 5 Gy. 4 h later, TC-EC adhesion was analyzed as described in Methods. **p≤0.01 (n = 8). C: Human colon carcinoma (HT29) and/or endothelial cells (HUVEC) were left untreated or were pretreated overnight with lovastatin (10 µM) (Lova). Afterwards, cells were irradiated (10 Gy) (IR) and TC-EC interactions were assayed after further incubation period of 4 h as described in Methods. See also Figure S1 and S2. ***p≤0.001; **p≤0.01 (n = 8). D: Logarithmically growing human endothelial (HUVEC) or tumor cells (HT29) were left untreated or were pretreated overnight with lovastatin (Lova). Afterwards cells were irradiated (IR) with 10 Gy and harvested 2 h or 4 h later for analysis of mRNA expression of various cell adhesion molecules. GAPDH mRNA expression was determined as internal loading control.

Previously, we reported a pro-adhesive IR response of human endothelial cells (EC), which is due to the upregulation of endothelial adhesion molecules, in particular E-selectin, via NF-κB [7]. Here, we expand on this observation showing that irradiation of HT29 human colon carcinoma cells (TC) increases their binding to human umbilical vein endothelial cells (HUVEC) *in vitro*. Tumor cell-endothelial cell (TC-EC) adhesion following irradiation of tumor cells occurred in a dose dependent manner (Fig. 1A). Irradiation of both tumor and endothelial cells exerted additive effects (Fig. 1B). Similar pro-adhesive effects were obtained after irradiation of tumorigenic rodent cells (i.e. CHO-K1 cells; see Figure S1). Whereas pre-treatment of HUVEC with the lipid lowering drug lovastatin attenuated TC-EC adhesion, pre-treatment of tumor cells with lovastatin was ineffective (Fig. 1C). This indicates that the anti-adhesive effect of lovastatin mainly rests on a reduced upregulation of endothelial adhesion factors following radiation exposure. Similar to IR, treatment of HT29 cells with the pro-inflammatory cytokine TNFα, which is another prototypical NF-κB activating agent, also augmented TC-EC adhesion (see Figure S2A). We also show that TC-EC adhesion following TNFα treatment of HUVEC [7], was further enhanced when the colon carcinoma cells were irradiated (see Figure S2B and S2C). Whether treatment of either HUVEC or HT29 cells with TNFα plus IR ameliorates TC-EC adhesion as compared to a single treatment remains to be tested. Regarding HUVEC, such additivity might be expected since we observed TNFα and IR to have additive effects on the expression level of E-selectin protein [7]. Overall, the data indicate that activation of the transcription factor NF-κB, either by TNFα or ionizing radiation, is of relevance for TC-EC adhesion.

### Lovastatin mitigates IR-induced expression of cell adhesion molecules in endothelial cells

To ascertain which cell adhesion molecules are most relevant for the IR-stimulated increase in TC-EC adhesion and the inhibitory effect of lovastatin, the mRNA expression levels of a subset of cell adhesion molecules were analyzed. In endothelial cells, IR increased the mRNA levels of E-selectin, VCAM and ICAM-1 (∼5.2-fold) (Fig. 1D) and IR-induced expression was largely blocked by lovastatin (Fig. 1D). We assume that protein expression is altered accordingly since we found in a previous study that changes in the mRNA levels of E-selectin and ICAM-1 are accompanied by congruent alterations in the amounts of protein [7]. In tumor cells, IR increased the mRNA expression of genes coding for CD44 (∼2.2-fold), LAMP2 (∼1.7-fold) and CXCR4 (∼3.1-fold), which are described as E-selectin specific counter-ligands [39], [40] (Fig. 1D). Notably, their mRNA expression level was not affected by lovastatin (Fig. 1D). This is in line with the observation that lovastatin pre-treatment of tumor cells did not impact TC-EC adhesion. Whether CD44, LAMP2 and/or CXCR4 are functionally involved in TC-EC adhesion stimulated by ionizing radiation is still unclear. Recently it has been reported that IR promotes the sialylation of integrin beta 1 [6]. Since sialylated proteins are discussed as E-selectin counter-ligands [25], it is conceivable that stimulation of TC-EC adhesion following irradiation of tumor cells might result from changes in the post-translational modification of pro-adhesive molecules rather than an increase in *de novo* protein synthesis. Overall, the data show that simultaneous irradiation of TC and EC has an additive effect on TC-EC adhesion *in vitro.* This is likely due to a concomitant upregulation of adhesion molecules in endothelial cells and corresponding counter-ligands in tumor cells.

### Ionizing radiation stimulates tumor cell extravasation and formation of lung metastases in mice

**Figure 2:**
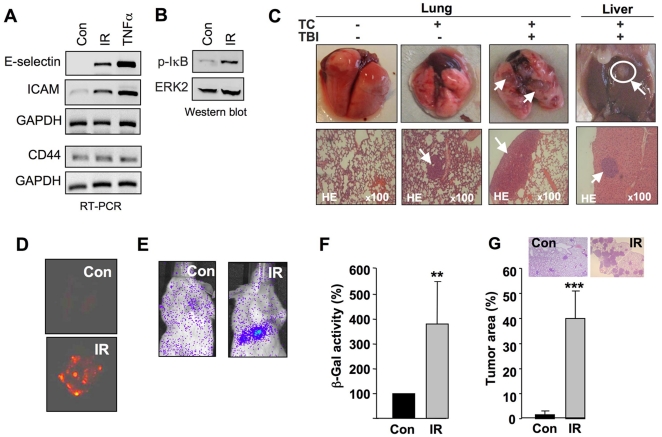
Total body irradiation triggers tumor cell extravasation and lung metastasis *in vivo.* A: Balb/c mice were irradiated (IR) with 6 Gy. 4 h after total body irradiation (TBI) mRNA expression of cell adhesion molecules was analyzed in large blood vessels from pulmonary and abdominal artery by RT-PCR. GAPDH mRNA expression was measured as internal loading control. As a positive control, isolated blood vessels were incubated with TNFα (10 ng/ml, 30 min, 37 ^o^C). B: Balb/c mice were subjected to TBI (6 Gy). 4 h after radiation (IR), phosphorylation of NF-κB inhibitor IκBα (p-IκBα), which is indicative of activation of NF-κB, was analyzed in liver extracts by Western blot analysis. ERK2 protein expression was determined as internal loading control. C: 2×10^6^ CHO-K1 cells were injected into the tail vein of immunodeficient Rag^2−/−^ Balb/c mice. Immediately after injection, mice were irradiated (4 Gy) (TBI). 3–4 weeks after TBI, the formation of metastases was analyzed in lung and liver. TC, tumor cells injected; TBI, total body irradiation. Shown are representative morphological and histopathological pictures (2–4 animals were analyzed per group). D: Red fluorescent protein overexpressing CHO-K1 cells were used for injection into the tail vein of immunodeficient Rag^2−/−^ Balb/c mice. Formation of lung metastases was analyzed 3–4 weeks after TBI with 4 Gy. Con, non-irradiated control; IR, TBI with 4 Gy. Shown are representative pictures (2–3 animals were analyzed per group). E: Four weeks after i.v. injection of luciferase overexpressing CHO-K1 cells followed by TBI (4 Gy), luciferase activity in the lung was monitored by use of a life imaging instrument as described in Methods. Shown are representative pictures (2–3 animals were analyzed per group). F: 3–4 weeks after i.v. injection of β-galactosidase (β-Gal) overexpressing CHO-K1 cells followed by TBI (4 Gy), relative β-Gal activity in lung extracts of irradiated mice was related to that of non irradiated control mice, which was set to 100%. **p≤0.01 (n = 4–8 mice). G: Formation of lung metastasis was quantitated in the lung of irradiated versus non-irradiated mice by calculating the % tumor area as related to the total lung area. Con, non-irradiated control; IR, TBI with 4 Gy. ***p<0.001 (n = 4–8 mice).

Binding of circulating tumor cells to the endothelium is a prerequisite for their extravasation *in vivo*. Hence, the *in vitro* data are alarming because they implicate that radiotherapy might promote metastasis *in vivo*. To scrutinize this hypothesis we performed experiments using different mouse models. We showed that total body irradiation (TBI) of Balb/c mice results in the upregulation of both E-selectin and ICAM-1 mRNA levels (Fig. 2A), which was accompanied by activation of NF-κB (Fig. 2B). This is fully in line with other experimental systems demonstrating activation of NF-κB and subsequent expression of E-selectin and ICAM mRNA and protein by IR [20], [34], [35], [41]. Next, we determined whether TBI promotes extravasation and metastasis. To this end, tumorigenic Chinese hamster (CHO-K1) cells, which were shown to have significant metastatic potential [42], were injected into the tail vein of immunodeficient mice (Rag^2−/−^ Balb/c strain). In our hands, CHO-K1 cells turned out to be the most appropriate cell line in this mouse model. Other cell lines tested were not metastatic at all or showed too high basal level of metastasis (see Figure S4). Immediately after injection, mice were subjected to TBI with a single dose of 4 Gy and colonization of the lung with metastases was analyzed 3–4 weeks later in comparison with non-irradiated animals. As shown in Fig. 2C, TBI caused a dramatic increase in the formation of lung metastases and, to a lesser extent, also increased the formation of liver metastases. Apparently, TBI increases the likelihood of extravasation of circulating tumor cells into the lung vasculature and subsequent formation of lung metastases. The pro-metastatic effect of TBI was further illustrated using CHO-K1 cells for transplantation that overexpress the red fluorescent protein (Fig. 2D) or the luciferase protein (Fig. 2E). Lung metastasis was also quantitated after intravenous injection of β-Gal overexpressing CHO-K1 cells followed by the analysis of β-galactosidase activity in protein extracts prepared from the lung of irradiated versus non irradiated mice. These studies revealed that TBI following transplantation of tumorigenic cells stimulated lung metastasis by about 4–5-fold (Fig. 2F). The pro-metastatic effect of TBI was also demonstrated by calculating the tumor area in lung sections, which was significantly enhanced as compared to non-irradiated mice (Fig. 2G). The data clearly show that TBI of mice harbouring circulating tumor cells leads to a massive colonization of the lung with metastases. Since extravasation of circulating tumorigenic cells is a prerequisite for the formation of lung metastases and, as shown above, TC-EC adhesion is stimulated by IR, we conclude that TBI promotes the process of tumor cell extravasation. With respect to the clinical situation, the data point to the possibility that the well established and highly appreciated therapeutic effect of radiotherapy (i.e. killing of tumor cells) might be influenced by an increased probability of circulating tumor cells, which have survived irradiation, to extravasate and develop tumors at secondary sites.

### Lovastatin counteracts IR-induced metastasis in mice

**Figure 3:**
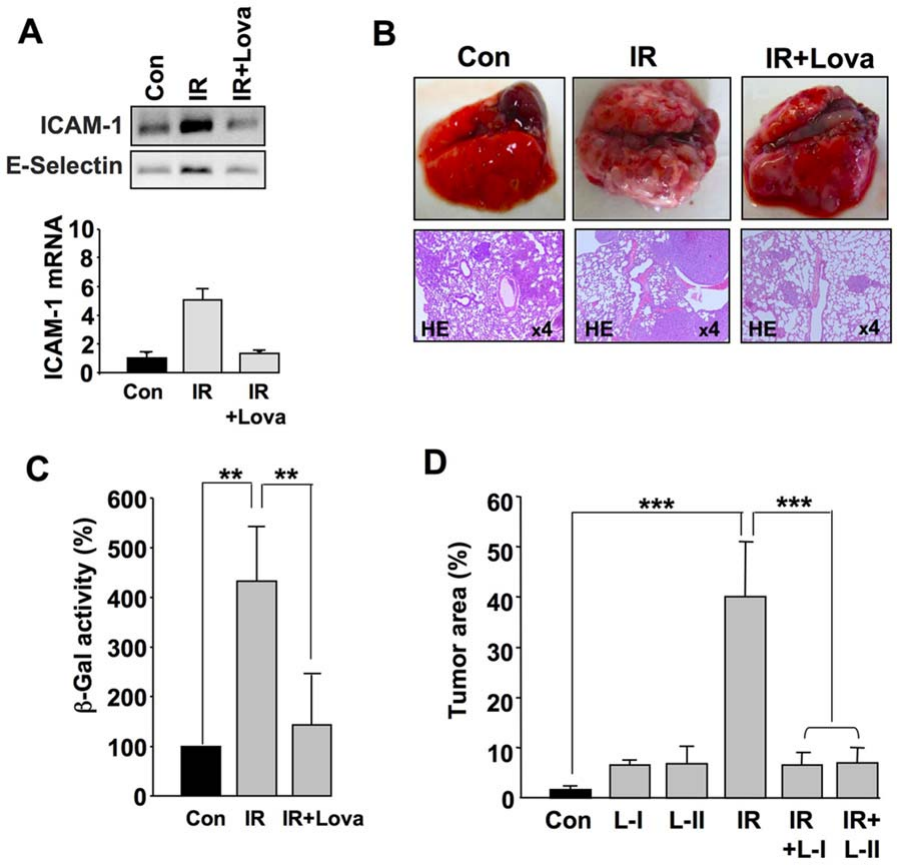
Lovastatin attenuates IR-induced metastasis *in vivo.* A: Balb/c mice were pretreated with lovastatin (10 mg/kg, p.o.) and irradiated with 6 Gy. 4 h after TBI, large blood vessels (i.e. pulmonary and abdominal artery) were isolated for total mRNA preparation and subsequent semiquantitative RT-PCR. ICAM-1 mRNA expression levels shown in the histogram were obtained by real-time PCR analyses (triplicate determinations). The level of ICAM-1 mRNA was normalized to the levels of GAPDH and β-actin and set to 1.0 in the untreated control (Con). Con, non-irradiated control; IR, irradiation; IR+Lova, IR exposure after lovastatin pre-treatment. B, C: 2×10^6^ CHO-K1 cells were injected into the tail vein of immunodeficient Rag^2−/−^ Balb/c mice which had been pre-treated or not with lovastatin (10 mg/kg, p.o.) for 2 days. Immediately after tumor cell injection, mice were irradiated with 4 Gy (IR). Post-treatment with lovastatin was not performed. 3–4 weeks after TBI, formation of metastasis was analyzed. B, IR, irradiation; IR+Lova, IR exposure after lovastatin pre-treatment. Shown are representative morphological and histopathological pictures (from n = 4–6 animals per group). C, The protective effect of lovastatin on IR-induced extravasation and lung metastasis of CHO-K1 cells was quantitated by determination of β-Gal activity in protein extracts from lung. Relative β-Gal activity in untreated control was set to 100%. See also Figure S3. **p≤0.01 (n = 4 mice). D: 2×10^6^ CHO-K1 cells were injected into the tail vein of immunodeficient Rag^2−/−^ Balb/c mice which had been pretreated or not with lovastatin for 14 days (10 mg/kg, p.o., 3x per week). Immediately after tumor cell injection, mice were irradiated with 4 Gy (IR). 3–4 weeks later, the percent tumor area in lung sections was calculated as described in methods. IR, irradiation; IR+Lova, IR exposure after lovastatin pre-treatment; L-I, lovastatin pre-treatment only; L-II, lovastatin pre- and post-treatment. Post-treatment (10 mg/kg, p.o., 3x per week) was performed for 2 weeks. *** p≤0.001 (n = 4 mice per group).

In search of drugs counteracting the putative pro-metastatic effect of IR, we explored the HMG-CoA reductase inhibitor lovastatin. The rationale behind this is that statins, which are widely used as lipid-lowering drugs, block the Rac1-regulated and NF-κB-dependent expression of E-selectin following TNFα or IR exposure of endothelial cells *in vitro* [7], [36]. Lovastatin mitigates TC-EC interaction *in vitro* (see Fig. 1) and inhibits activation of NF-κB following irradiation *in vitro* [7] and *in vivo* [43]. Moreover, statins alleviate normal tissue damage (i.e. pro-inflammatory and pro-fibrotic radiation responses) that results from radiotherapy as acute or delayed side effect [44], [45], [46], [47]. In agreement with our *in vitro* data, lovastatin impaired the TBI-induced upregulation of cell adhesion molecules *in vivo* (Fig. 3A). Most importantly, short-time pre-treatment of Rag^2−/−^ Balb/c animals for 2 days with lovastatin clearly attenuated the TBI-induced formation of lung metastases (Fig. 3B). We should note that lovastatin does not increase the sensitivity of CHO-K1 cells to irradiation [48]. Assaying β-galactosidase activity in lung extracts, which was taken as indication of the tumor burden, we calculated that lovastatin reduced the TBI-stimulated formation of lung metastases by about 75% (Fig. 3C). Amazingly, under these experimental conditions of short-time pre-treatment with lovastatin, the statin had a weak prometastatic effect on its own (see Figure S3). Extending the pre-treatment period with lovastatin for 14 days, the prometastatic statin effect was largely vanished and TBI-stimulated lung metastasis was completely blocked (Fig. 3D). Pre- and post-treatment with lovastatin had the same anti-metastatic effect as pre-treatment alone (Fig. 3D), showing that (i) pretreatment with lovastatin is sufficient to block metastasis and (ii) post-treatment with lovastatin for an extended period of time does not result in adverse effects with respect to metastasis.

**Figure 4:**
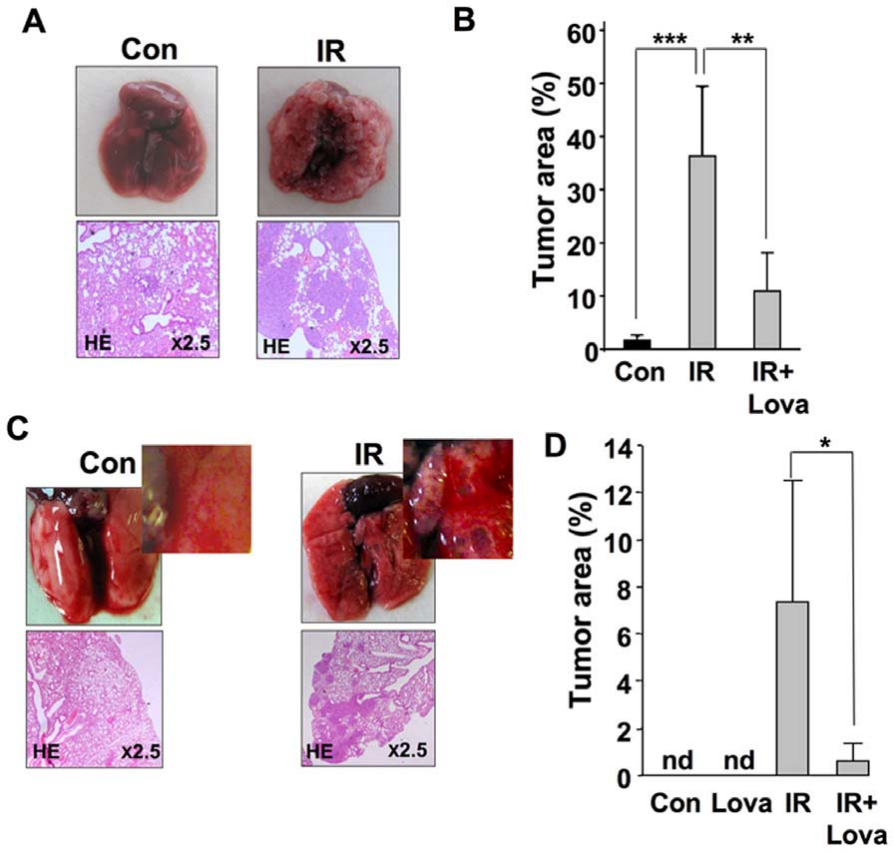
Radiation response of normal tissue is sufficient for provoking lung metastasis. A, B: Rag^2−/−^ Balb/c mice that have been pre-treated or not with lovastatin (Lova) for 4 days (10 mg/kg, p.o.) were irradiated with 4 Gy before tumorigenic (non-irradiated) CHO-K1 cells (2×10^6^) were injected into the tail vein. 3–4 weeks later, formation of lung metastases was analyzed. A, representative morphological and histopathological pictures demonstrating the pro-metastatic effect of TBI; B, quantitative analysis of tumor area in lung sections. Con, non-irradiated control; IR, TBI with 4 Gy. ** p≤0.01; *** p≤0.001 (n = 4–8 mice). C, D: 2×10^6^ human colon carcinoma cells (HT29) were injected into the tail vein of CB-17 SCID mice which have been pretreated or not with lovastatin (Lova) for 4 days (10 mg/kg, p.o.). Immediately after injection of the tumor cells, animals were irradiated with 2.5 Gy. 3–4 weeks later, lung metastases were analyzed by calculating the tumor area as described in Methods. C, representative morphological and histopathological pictures illustrating the IR effect; D, quantitative analysis of percent (%) tumor area in lung sections. Con, non-irradiated control; IR, total body irradiation with 2.5 Gy; nd, no tumor cells detectable. * p≤0.05 (n = 3–4 mice). See also Figure S4.

In order to elucidate whether TBI was effective in stimulating metastasis when the animals, but not the tumor cells were irradiated, mice were irradiated first and thereafter tumor cells were transplanted. As shown in Fig. 4A, TBI of animals followed by transplantation of non-irradiated tumor cells also resulted in enhanced formation of lung metastases as compared to mice that have not been pre-irradiated. When the animals were pre-treated with lovastatin before they were subjected to TBI and then received non-irradiated tumor cells, the formation of lung metastases was reduced (Fig. 4B). These findings show that the pro-metastatic effect of TBI is a trans-effect, i.e. it is due to a radiation response of the normal tissue that stimulates the metastatic properties of the tumor cells. It is reasonable to suggest that this occurs via transient activation of pro-adhesive endothelial functions. Pre-treatment of mice with lovastatin is sufficient to block this pro-adhesive radiation response of the endothelium and, therefore, antagonizes IR-mediated extravasation of tumor cells and formation of lung metastases.

Whether TBI promotes lung metastasis of human tumor cells could not be addressed in the Rag^2−/−^ Balb/c mouse model since none of the human tumor cell lines tested (see Figure S4) formed lung metastases in this host. Therefore, we transplanted human HT29 colon carcinoma cells into CB-17 SCID mice where they formed lung metastases with a low basal frequency. Because SCID mice are radiosensitive, which is due to a defect in DNA repair [49], it was necessary to irradiate the animals with lower IR doses. It can be assumed that this dose reduction has a negative impact on the pro-metastatic response of the animals and, therefore, the SCID-based model was applied for confirmatory purpose only. Despite these limitations, the results fully confirmed the data obtained with the CHO-K1/Rag^2−/−^ system. Thus, we observed that TBI with 2.5 Gy promotes lung metastasis after i.v. injection of HT29 cells into CB-17 SCID mice (Fig. 4C). Moreover, lovastatin pre-treatment largely neutralized TBI-stimulated formation of lung metastases in the HT29 cells/SCID mouse model (Fig. 4D). Taken together, we were able to show using two experimental *in vivo* model systems (i.e. rodent CHO-K1 cell/Rag^2−/−^ Balb/c mice and human HT29 cell/CB-17 SCID mice) that TBI stimulates the extravasation of circulating tumor cells and subsequent formation of lung metastases and that this effect is attenuated by lovastatin. The *in vivo* data thus support the notion of a pro-metastatic potency of IR and the benefit of lovastatin in blocking extravasation and metastasis.

### E-selectin is involved in IR-induced tumor cell extravasation and metastasis

**Figure 5:**
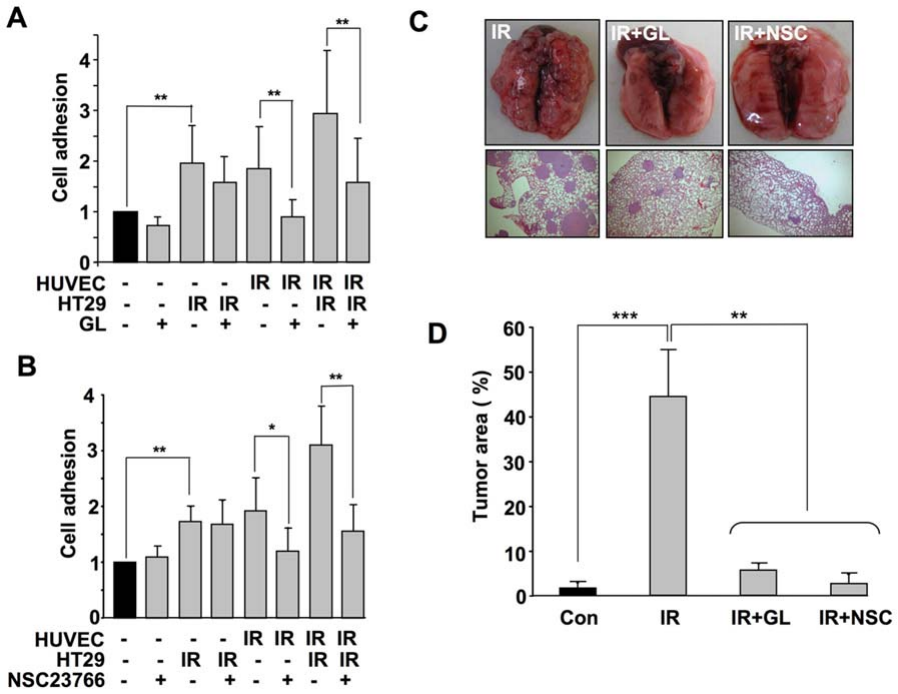
The small GTPase Rac1 and E-selectin ligands are involved in IR-induced TC-EC adhesion *in vitro* and TBI driven extravasation of metastasis *in vivo*. A, B: Human colon carcinoma cells (HT29) and/or endothelial cells (HUVEC) were left untreated or were pre-treated with the Sialy-Lewis X mimetic glycyrrhizic acid (2 mM, 30 min) (A) or the Rac1 inhibitor NSC23766 (100 µM, 4 h) (B). 4 h after irradiation (10 Gy), TC-EC adhesion was analyzed as described in methods. *p≤0.05; **p<0.01 (n = 8). C, D: CHO-K1 cells (2×10^6^) were injected into the tail vein of Rag^2−/−^ Balb/c mice that have been pre-treated or not with glycyrrhizic acid (GL), NSC23766 (NSC). 3–4 weeks after TBI (4 Gy), formation of lung metastases was analyzed. C, Representative morphological and histopathological photographs are shown. D, The histogram displays the percent (%) tumor area in lung sections. Con, non-irradiated control; IR, TBI with 4 Gy. ** p≤0.01; *** p≤0.001 (n = 4–8 mice).

Sialyl-Lewis X structures are known as major counter-ligands of the endothelial adhesion molecule E-selectin [25], [50]. They have been shown to be of importance for the progression of colon cancer [23]. To examine whether these tetrasaccharide carbohydrates are involved in IR-stimulated TC-EC adhesion *in vitro* and metastasis *in vivo*, we made use of the Sialyl-Lewis X mimetic glycyrrhizic acid (GL) [51], which is the major sweet tasting compound of liquorice root. As shown in Fig. 5A, glycyrrhizic acid largely blocked IR-induced binding of tumor cell to endothelial cells, indicating that E-selectin and its ligand Sialyl-LewisX are important for radiation-promoted TC-EC adhesion *in vitro*. Next, we investigated the involvement of the E-selectin counter-ligand Sialyl-Lewis X on TBI-driven extravasation and metastasis *in vivo*. Fully in line with our *in vitro* data we observed that pre-treatment of mice with GL largely attenuated the TBI-induced extravasation and formation of lung metastases (Fig. 5C). The data support the concept that E-selectin and its ligands are essential for TBI-stimulated tumor cell extravasation and metastasis.

### The Ras-homologous small GTPase Rac1 is required for IR-driven pro-metastatic processes

The E-selectin gene is known to be regulated by the transcription factor NF-κB [35]. As mentioned above, the Ras-homologous GTPase Rac1 is required for the activation of NF-κB by TNFα and IR [7], [36]. Therefore, it is reasonable to speculate that Rac1 signaling might interfere with the radiation-induced TC-EC interaction *in vitro* and metastasis *in vivo*. In line with this, we found that pharmacological inhibition of Rac1 by NSC23766 [52] impairs the radiation-stimulated increase in TC-EC adhesion *in vitro* (Fig. 5B). Most importantly, pre-treatment of mice with NSC23766 also blocked TBI-induced tumor cell extravasation and formation of lung metastases *in vivo* (Fig. 5C and Fig. 5D). Therefore, we suggest that TBI-induced extravasation of circulating tumor cells rests on Rac1/NF-κB-dependent upregulation of endothelial adhesion molecules, in particular E-selectin, and involves tumor specific E-selectin ligands such as Sialyl-LewisX carbohydrates. Since Rho GTPases, including Rac1, are known as highly relevant targets of statins, we speculate that the observed protective effect of lovastatin against TBI-induced extravasation and metastasis rests on the inhibition of Rac1-NF-κB-regulated upregulation of E-selectin.

**Figure 6:**
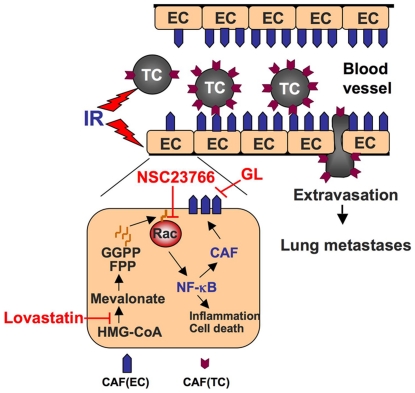
Model of IR-induced tumor cell extravasation and metastasis and pharmacological strategies to prevent this adverse radiation effect. Ionizing radiation (IR) triggers the expression of different types of cell adhesion molecules on tumor (TC) and endothelial (EC) cells, which augment TC-EC adhesion and subsequent extravasation of tumor cells. By inhibiting Rac1-regulated activation of NF-κB, HMG-CoA reductase inhibitors (statins) specifically attenuate IR-induced expression of endothelial cell adhesion molecules, thereby antagonizing radiation-promoted adhesion of circulating tumor cells as well as subsequent extravasation and formation of lung metastases. TBI-promoted metastasis can also be reduced by targeting of the small GTPase Rac1 using the small molecule inhibitor NSC23766 and/or the Sialyl-Lewis X mimetic glycyrrhizic acid, which acts as E-selectin antagonist. CAF, cell adhesion factors; FPP, farnesylpyrophosphate; GGPP, geranylgeranylpyrophosphate; GL, Glycyrrhizic acid.

In summary, we provide evidence that IR largely stimulates EC-TC adhesion *in vitro* and that whole body irradiation of mice increases the probability of extravasation and subsequent formation of lung metastases of transplanted circulating tumorigenic cells of rodent and human origin. The pro-metastatic radiation effect appears to rest on the upregulation of both endothelial and tumor cell-specific adhesion factors (see Fig. 6). Importantly, the radiation response is a trans-effect since irradiation of mice prior to transplantation of non-irradiated tumor cells also stimulated metastasis. From this we infer that irradiation of the normal tissue is sufficient to trigger metastasis. Searching for pharmacological strategies counteracting the adverse radiation effect, we show that pre-administration of lovastatin, which is nowadays widely used as lipid-lowering drug, can antagonize pro-adhesive radiation effects *in vitro* and the pro-metastatic effects of TBI *in vivo*. The anti-metastatic statin effect is very likely due to inhibition of IR-induced normal tissue responses, in particular the IR-stimulated Rac-1-regulated increase in the expression of endothelial cell adhesion molecules (see Fig. 6). Thus, the proposed pharmacological strategy suitable to counteract pro-metastatic effects of RT is based on the inhibition of Rac1 signaling, either by statins (e.g. lovastatin) or Rac1-specific inhibitors, such as NSC23766 (see Fig. 6). Alternatively, radiation-stimulated binding of tumor cells to the endothelium can be blocked by Sialyl-LewisX mimetic drugs, which act as E-selectin antagonists (see Fig. 6). However, neither Sialyl-LewisX mimetics nor Rac1 inhibitors have thus far been established for clinical use. Therefore, we propose to focus on statins that are clinically well established, very well tolerated and widely used for lipid lowering purpose. We consider them as first choice drugs for short-term clinical application.

Regarding speculations about the clinical relevance of the findings, it is important to consider the question whether the local concentration of circulating tumor cells in the lung vasculature is high enough to achieve significant extravasation in humans, in particular because radiation to the lung is kept to a minimum in the clinic. Besides, although extravasation is essential for metastasis, additional factors (e.g. motility, proliferation, vascularization) are required to complete the formation of metastases. Despite the complexity of the metastatic process, the data support the concern raised in previous reports [11], [12], [14], [16], [18] that RT might stimulate metastasis. This notion should not be taken as an argument against the well-known therapeutic benefit of RT. It just raises the point that, if tumor cells are not fully eradicated by RT, there is the possibility of an increased level of extravasation and metastasis in the survivors. This also pertains to prophylactic irradiation. Although there is no clinical evidence that prophylactic irradiation increases metastasis, a pro-metastatic potential cannot be excluded. Thus, the data reported here support a previous note that calls for a critical reassessment of the benefit of prophylactic irradiation [17], [53]. Apparently, further preclinical studies are required using clinically more appropriate experimental settings, for example fractionated local irradiation of the lung with low doses. Irrespective of this, we propose on the basis of the data that the inclusion of statins into current radiotherapeutic regimen is a reasonable strategy for improving the long-term clinical outcome of radiation-based tumor therapy. Since statins are widely used for lipid lowering purposes, the question regarding the clinical relevance of our data might easily be addressed in retrospective and/or prospective clinical studies in which statins are administered concomitantly with RT.

## Materials and Methods

### Materials

For treatment of cells and animals with ionizing radiation (i.e. γ-rays), a ^60^Co source (Atomic Energy of Cananda Ltd.) was used. Total RNA was isolated by use of E.Z.N.A. Total RNA Kit (Omega Bio-Tek, Norcross, USA). cDNA synthesis was performed using iScript^TM^ cDNA Synthesis Kit (Biorad, Munic, Germany). TNFα, Calcein AM solution, glycyrrhizic acid and DNA oligos originate from SIGMA-Aldrich (Taufkirchen, Germany). dNTPs, the PCR Y-buffer, and nitrocellulose were from Perkin Elmer (Rodgau, Germany). Orange Loading Dye Solution was obtained from Fermentas (St. Leon-Rot, Germany). The Absolute QPCR SYBR Green Fluorescein Mix was from Thermo Fisher (Schwerte, Germany). ERK2 antibody used in this study was obtained from Santa Cruz (Heidelberg, Germany), antibody for phosphorylated IκBα (p-IκBα) originated from New England Biolabs (Frankfurt am Main, Germany). Hyperfilm ECL was from GE Healthcare (München, Germany). Taq DNA Polymerase was a kind gift of H. Kleinert (Mainz, Germany). Lovastatin and Rac1 inhibitor NSC23766 were purchased from Calbiochem-Novabiochem (Bad Soden, Germany) and dissolved in ethanol for the in vitro analyses. For the *in vivo* studies lovastatin was solubilized in 0.9% saline and sonicated prior to oral administration or injection. Primary cultures of human umbilical vein endothelial cells (HUVEC) were obtained from Lonza Sales Ltd (Basel, CH).

### Cell culture conditions

Cells of rodent origin (CHO-K1 hamster fibroblasts [54] and B16F10 mouse melanoma cells [55]) and human tumor cells (HT29 colon carcinoma cells [56], MCF-7 and T47D mammary carcinoma cells [48]) were grown in RPMI medium containing 10% fetal bovine serum (FBS) supplemented with penicillin and streptomycin. Human DLD-1 colon carcinoma cells (originating from the American Type Culture Collection (ATCC)) were cultured in DMEM plus 10% FBS. Primary human umbilical vein endothelial cells (HUVEC) (purchased from Cambrex (Bio Whittaker Europe)) were cultured in endothelial cell growth media system (EGM-2; Lonza Ltd, Basel, CH) containing 2% FCS, 0,04% hydrocortisone, 0,4% hFGF-B, 0,1% VEGF, 0,1% R^3^-IGF-1, 0,1% Ascorbic acid, 0,1% hEGF, 0,1% GA-1000 and 0,1% heparin). HUVEC were used in the 4th to 10th passage for the experiments. Cultures were kept at 37°C in humidified atmosphere containing 5% CO_2_.

### Total RNA purification and RT-PCR reaction

1 µg of purified total RNA was used for cDNA synthesis. Reverse transcription was performed at 25°C for 5 min, 42°C for 30 min and 85°C for 5 min. For PCR reaction 30 cycles were performed (denaturation: 94°C, 1 min; annealing: 60°C, 2 min; polymerization: 68°C, 2 min). PCR products were separated onto agarose gels and DNA was visualized by ethidium bromide staining. The sequence of the sense (s) and antisense (as) primers used for amplification reactions were as follows: E-selectin, f: TCTCTCAGCTCTCACTTTG, r: TTCTTCTTGCTGCACCTCT (384 bp); VCAM, f: AATTTATGTGTGTGAAGGAG, r: GCATGTCATATTCACAGAA (466 bp); ICAM, f: GGCTGGAGCTGTTTGAGAAC, r: ACTGTGGGGTTCAACCTCTG (202 bp); LAMP-2, f: GGTTAATGGCTCCGTTTTCA, r: ATGGGCACAAGGAAGTTGTC (216 bp); CD44, f: AGAAGGTGTGGGCAGAAGAAAA, r: CATTCTGCAGGTTCCTTGTCT (187 bp); TIMP, f: ATTCCGACCTCGTGATCAG, r: CGTCCACAAGCAATGAGTG (405 bp); CXCR4, f: GGTGGTCTATGTTGGCGTCT, r: TGGAGTGTGAC AGCTTGGAG (227 bp); CXCL12, f: CTTTAGCTTCGGGTCAATGC, r: TCAGCCTGAGCTACAGATGC (161 bp); b_1_-Integrin, f: CGAGGTCATGGTTCATGTTG, r: CAGTGTTGTGGGATTTGCAC (294 bp); b_3_-Integrin, f: GACAAGGGCTCTGGAGACAG, r: ACTGGTGAGCTTTCGCATCT (231 bp); GAPDH, f: GAAGATGGTGATGGGATTTC, r: GAAGGTGAAGGTCGGAGTC (313 bp). Real-time PCR was performed using the Absolute QPCR SYBR Green Fluorescein Mix (Thermo Fisher) and a MyIQ Thermal Cycler (BioRad). For each reaction, 2 µl of diluted (1∶10) cDNA and specific primers (0,06 µM each) were used. After denaturation step (95°C, 15 min), PCR reaction (40 cycles) was conducted according to the following protocol∶95°C, 30 sec; 60°C, 1 min; 72°C, 1 min. At the end of each reaction, the melting curve was recorded to ensure the specificity of the reaction. From each sample, real-time PCR analysis was performed in duplicate or triplicate. Data were analyzed using IQ5 Optical System Software 2.0 (BioRad). The sequence of the sense (s) and antisense (as) primers used for amplification reactions were created using the Primer3 software. GAPDH, f: AACTTTGGCATTGTGGAAGG, r: CACATTGGGGGTAGGAACAC (222 bp product); b-actin, f: GCATTGCTGACAGGATGCAG, r: CCTGCTTGCTGATCCACATC (159 bp); E-selectin, f: AGCTACCCATGGAACACGAC, r: ACGCAAGTTCTCCAGCTGTT (199 bp); ICAM-1, f: CGAAGGTGGTTCTTCTGAGC, r: GTCTGCTGAGACCCCTCTTG (238 bp), CD44, f: TGGATCCGAATTAGCTGGAC, r: AGCTTTTTCTTCTGCCCACA (189 bp).

### Preparation of protein extracts, SDS PAGE and Western Blot analysis

For preparation of total protein extracts 15 mg of the respective organs were disrupted by Ultra-Turrax in lysis buffer (20 mM TRIS, pH 7,5, 150 mM NaCl, 1 mM EDTA, 1 mM EGTA, 2,5 mM sodium pyrophosphate, 1 mM Na_3_VO_4_, 1 µg/ml Complete EDTA free (Roche), 1 mM PMSF, 1% Triton X-100). Subsequently, samples were sonicated. After centrifugation (10 min, 10.000 x g, 4 ^o^C), the pellet was discarded and the supernatant used for protein determination by Bradford and subsequent Western Blot analysis. Protein extracts were separated by SDS PAGE. Subsequently, proteins were transferred onto nitrocellulose membranes (Perkin Elmer) using a Protean Mini Cell (BioRad). After completion of the transfer, membranes were blocked in 5% non-fat milk in TBS/0.1% Tween 20 for at least 60 min. Incubation with the primary antibody (as indicated) was conducted overnight at 4°C. Incubation with peroxidase conjugated anti-mouse or anti-rabbit secondary antibody (1∶2000) (Rockland) was performed for 60 min at room temperature. Bound antibodies were then visualized using a chemiluminescence reaction and Hyperfilm ECL (GE Healthcare). For densitometrical quantification of the autoradiographies, the Multi Analyst software (BioRad) was applied.

### Analysis of cell adhesion

Tumor cell - endothelial cell adhesion (TC-EC adhesion) was analyzed by an ELISA-based method as described [7]. Briefly, cells were pretreated or not with lovastatin (10 µM) for 16h before TNFα treatment or irradiation was performed. Tumor cells were labeled with the fluorescent dye Calcein AM (5 µM, 30 min, 37°C) in RPMI containing 10% FCS. Cells were washed with PBS and resuspended in RPMI containing 10% FCS. 5×10^5^ labeled cells were added to confluent monolayers of HUVEC (human umbilical vein endothelial cells), which have been stimulated or not 4 h earlier with TNFα or γ-rays, for 2 h at 37°C. After removal of nonadherent cells by washing with PBS twice, the attached cells were quantified by fluorimetric analysis (excitation wavelength: 495 nm, emission: 517 nm). Relative adhesion of non-stimulated tumor cells to non-stimulated endothelial cells was set to 1.0. Data are shown as mean values ± SD from 2–4 independent experiments each performed in quadruplicate (n≥8). For statistical evaluation Students t-test was used.

### *In vivo* analyses

For *in vivo* experiments, immunodeficient Rag^2−/−^ Balb/c mice (both provided by our local animal facility) were used. Mice were bred in our local specific-pathogen free animal housing facility and were 3–4 months of age at the start of the experiments. To analyze the effect of lovastatin on early radiation responses, Balb/c mice were pre-treated with lovastatin p.o. (10 mg/kg BW) for 2 days. At day 3 the mice were irradiated with 6 Gy and sacrificed 4 h or 24 h later. Blood vessels (isolated from pulmonary and abdominal artery) and organs were isolated for the analysis of mRNA and protein expression. For analyzing the effect of total body irradiation (TBI) on the extravasation and lung colonization of circulating cells the CHO-K1 system, which is an accepted model of metastasis [42], was routinely used. Cells were left untransfected or were stably transfected with either a β-Gal (pcDNA3.1His/lacZ (Invitrogen, Karlsruhe, Germany)), luciferase expression vector (pGL2-basic (Promega, Mannheim, Germany)) or pTurboFP635-C Vektor (Evrogen, Moscow, Russia) and injected into the lateral tail vein of immunodeficient Rag^2−/−^ Balb/c mice (2×10^6^ cells/0.25 ml PBS/mouse). Lovastatin pre-treatment of the mice (10 mg/kg, p.o. or i.p.) was performed for different period of time (2d – 14d). Immediately after injection of the tumor cells, animals were irradiated (total body irradiation) with 4 Gy. TBI was used as a proof-of-principle approach to assess the role of radiation on metastasis. 4 weeks later animals were sacrificed and organs, in particular the lung, were examined macroscopically for the presence of metastases. Lung tissue from each animal as well as any other tissue containing macroscopically detectable metastases were fixed in formaldehyde or frozen in liquid nitrogen for pathological examinations and biochemical analyses, respectively. Glycyrrhizic acid was applied twice, i.e. 24 h (10 mg/kg; i.p.) and 0.5 h (5 mg/kg; i.p.) before TBI. The Rac inhibitor NSC23766 (5 mg/kg; i.p) was given 48 h and 24 h before TBI. Data shown are mean values ± SD based on the results of at least 2–4 independent experiments with 2–4 animals per group in each experiment (total number of animals 4–8). Statistical significance was calculated using Students t-test. For confirmation of the results, a second independent model system, consisting of immunodeficient CB-17 SCID mice that were irradiated with 2.5 Gy after injection of human colon carcinoma cells (HT29), was used. Under our experimental conditions, the mice behaved normal throughout the experiment and did not show major signs of toxicity such as loss of weight or scrubby coat. All animal work performed in this study was conducted according to the national guidelines and was reviewed and approved by an institutional review board/ethics committee headed by the local animal welfare officer (Prof. Kempski) of the University Medical Center (Mainz, Germany). The animal experiments were additionally approved by the responsible national authority, which is the National Investigation Office Rheinland-Pfalz (Koblenz, Germany). The Approval ID assigned by this authority is the following: AZ 23177-07/G09-1-023.

### Histology and quantitative assessment of lung metastasis

Formaldehyde-fixed paraffin-embedded samples were cut into sections of 4 µm thickness and stained with haematoxylin and eosin (HE staining). To quantify the amount of metastases the expression of β-galactosidase activity was quantified in lung extracts using the Beta*-*Glo assay (Promega, Mannheim, Germany). To this end, lung tissue was homogenized with an ultraturrax and sonicated in sonication buffer (20 mM Tris-HCl, pH 8,5, 1 mM EDTA, 1 mM β-mercaptoethanol and 5% glycerol). Cell debris was removed by centrifugation (10′, 10000xg, 4°C). Supernatant was used for measuring the protein concentration according to Bradford before Beta-Glo Assay was performed according to the manufactureŕs protocol (Promega, Mannheim, Germany). In the reaction, 6-O-β-galactopyranosyl-luciferin serves as a substrate which is cleaved by β-galactosidase to yield luciferin which is metabolized by luciferase. Bioluminescence was detected using a luminometer (Berthold detection systems, Pforzheim, Germany). The β-gal activity was expressed as relative light units (RLU) and was set to 100% in non-irradiated controls. The fraction of tumor tissue in the lung was also quantitated in HE-stained lung sections (≥3 sections per lung/animal) by calculating the percentage of tumor tissue in relation to the normal tissue by use of imaging technique (Photoshop 7.0).

### *In vivo* imaging

Luciferase overexpressing CHO-K1 cells (2×10^6^) were injected into the lateral tail vein of Balb/c Rag^2−/−^ mice. 4 weeks after TBI with 4 Gy, 100 µl of luciferin solution (15 mg/ml in PBS (Promega)) was injected intraperitoneally. Ten minutes later mice were anesthetized by isoflurane and placed ventral side up in an IVIS Lumina II live image instrument (Caliper, Rüsselsheim, Germany). Images of luciferase activity were continuously acquired until maximal activity was detected. The acquired images were analyzed using the Living Image 3.0 software.

## Supporting Information

Figure S1 **IR stimulates the adhesion of CHO-K1 cells to HUVEC.** Chinese hamster ovary cells (CHO-K1) and/or endothelial cells (HUVEC) were left untreated or were pretreated overnight with lovastatin (10 µM) (Lova). Afterwards, cells were irradiated (10 Gy) (IR) and TC-EC interactions were assayed after further incubation period of 4 h as described in Methods.^1^irradiation after lovastatin pretreament. ** p≤0.01 (n = 4).
(TIF)

Figure S2 **Ionizing radiation promotes TNFα-provoked TC-EC adhesion.** A: TNFα treatment of both tumor cells (HT29) or endothelial cells (HUVEC) promotes TC-EC adhesion. Pretreatment with TNFα (10 ng/ml) was performed for 4 h. *p≤0.05; **p≤0.01 (n = 8). B, C: Endothelial cells (HUVEC) were left untreated or were treated with TNFα (10 ng/ml) for 4 h. Human colon carcinoma cells (HT29 (B) or DLD-1 (C)) or were irradiated with 2–10 Gy. 4 h after irradiation, tumor cells were added to the TNFα pre-treated monolayer of HUVEC and cell adhesion was measured as described in Methods. *p≤0.05; **p≤0.01 (n = 9–15).
(TIF)

Figure S3 **Effect of lovastatin on the formation of lung metastases.** 2×10^6^ CHO-K1 cells were injected into the tail vein of Rag^2−/−^ BALB/c mice which had been pretreated with lovastatin p.o. or i.p. for different periods of time. The formation of lung metastases was analyzed three weeks later. +, weak effect; ++, stronger effect. As further control, physiological NaCl solution was administered p.o. Data shown are based on the morphological analysis of n = 3–4 animals per experimental condition.
(TIF)

Figure S4 **Cell line specificity of formation of lung metastases.** 2×10^6^ cells were injected into the tail vein of Rag^2−/−^ BALB/c mice. Afterwards mice were irradiated with 4 Gy (total body irradiation). The formation of lung metastases was analyzed three weeks later. Control, non-irradiated; IR, total body irradiation; -, no lung metastases detectable; +, weak effect; +++, strong effect. Data shown are based on the morphological analysis of n = 3–4 animals per cell line used.
(TIF)
