# Supplementary figures and images for: Rac1-Regulated Endothelial Radiation Response Stimulates Extravasation and Metastasis That Can Be Blocked by HMG-CoA Reductase Inhibitors
Source: PLoS One. 2011 Oct 19;6(10):e26413. doi: 10.1371/journal.pone.0026413 (PMC3198428; doi:10.1371/journal.pone.0026413)

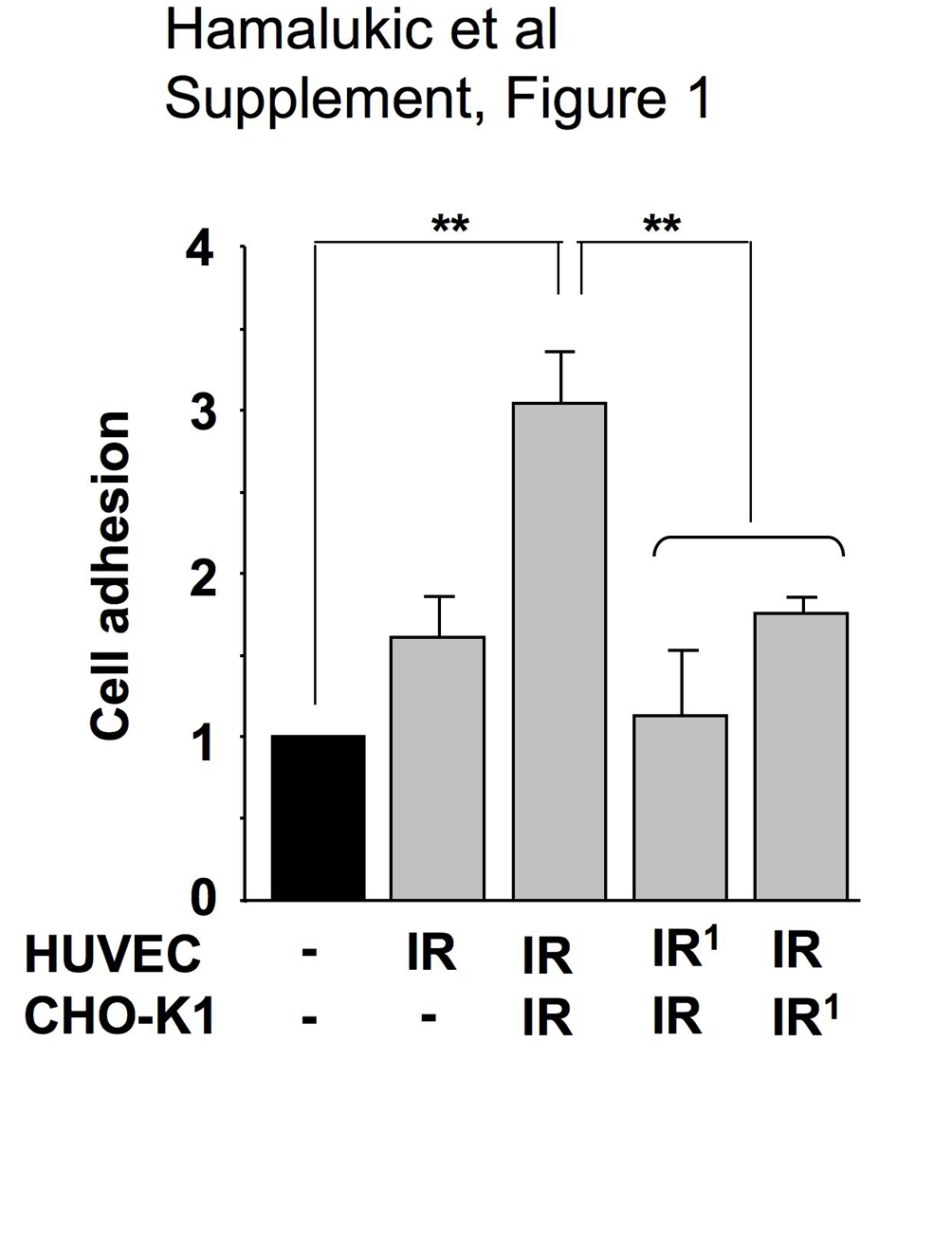

Supplement: Figure S1 — IR stimulates the adhesion of CHO-K1 cells to HUVEC. Chinese hamster ovary cells (CHO-K1) and/or endothelial cells (HUVEC) were left untreated or were pretreated overnight with lovastatin (10 µM) (Lova). Afterwards, cells were irradiated (10 Gy) (IR) and TC-EC interactions were assayed after further incubation period of 4 h as described in Methods.1irradiation after lovastatin pretreament. ** p≤0.01 (n = 4). (TIF) [file pone.0026413.s001.tif]

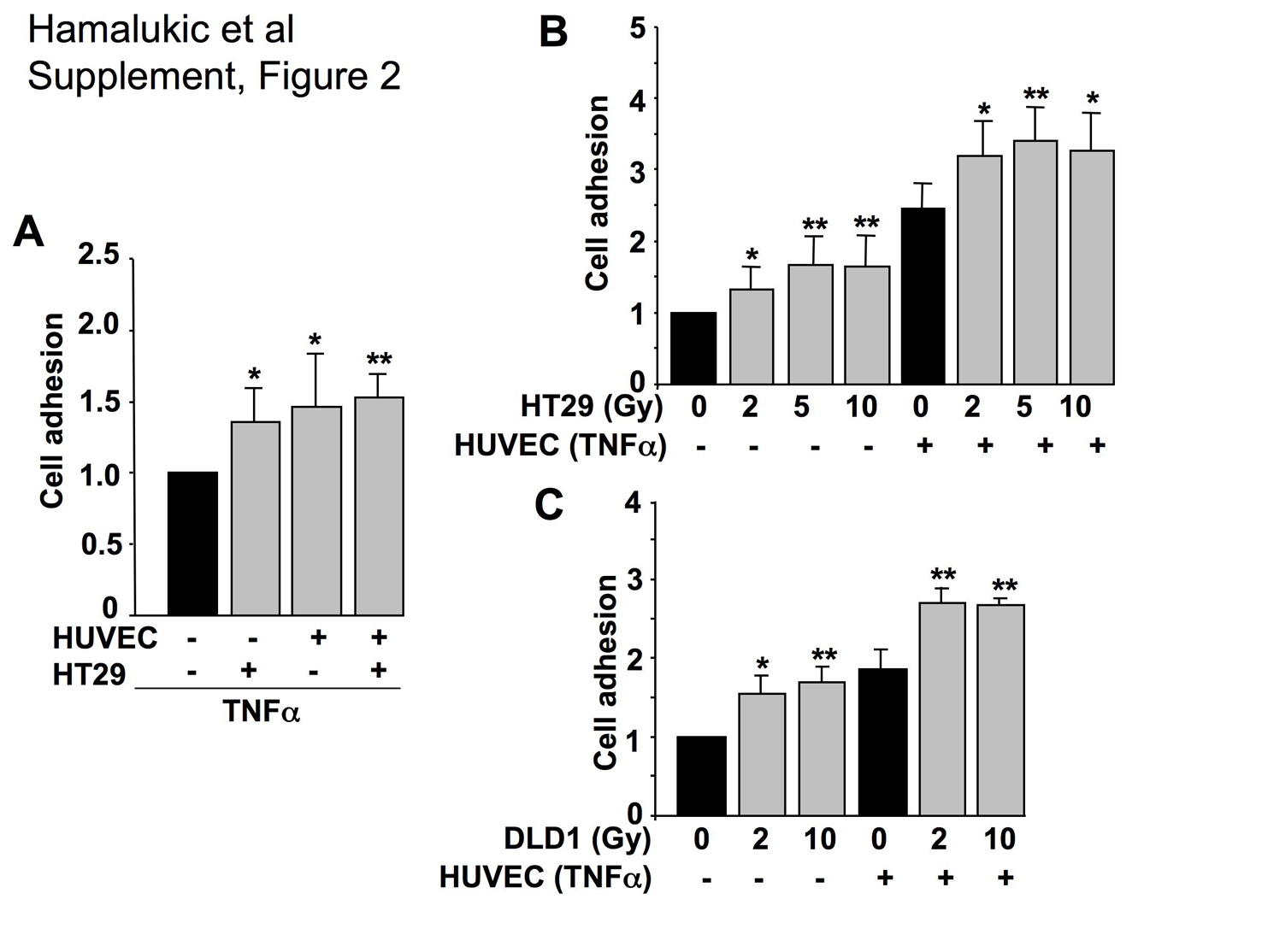

Supplement: Figure S2 — Ionizing radiation promotes TNFα-provoked TC-EC adhesion. A: TNFα treatment of both tumor cells (HT29) or endothelial cells (HUVEC) promotes TC-EC adhesion. Pretreatment with TNFα (10 ng/ml) was performed for 4 h. *p≤0.05; **p≤0.01 (n = 8). B, C: Endothelial cells (HUVEC) were left untreated or were treated with TNFα (10 ng/ml) for 4 h. Human colon carcinoma cells (HT29 (B) or DLD-1 (C)) or were irradiated with 2–10 Gy. 4 h after irradiation, tumor cells were added to the TNFα pre-treated monolayer of HUVEC and cell adhesion was measured as described in Methods. *p≤0.05; **p≤0.01 (n = 9–15). (TIF) [file pone.0026413.s002.tif]

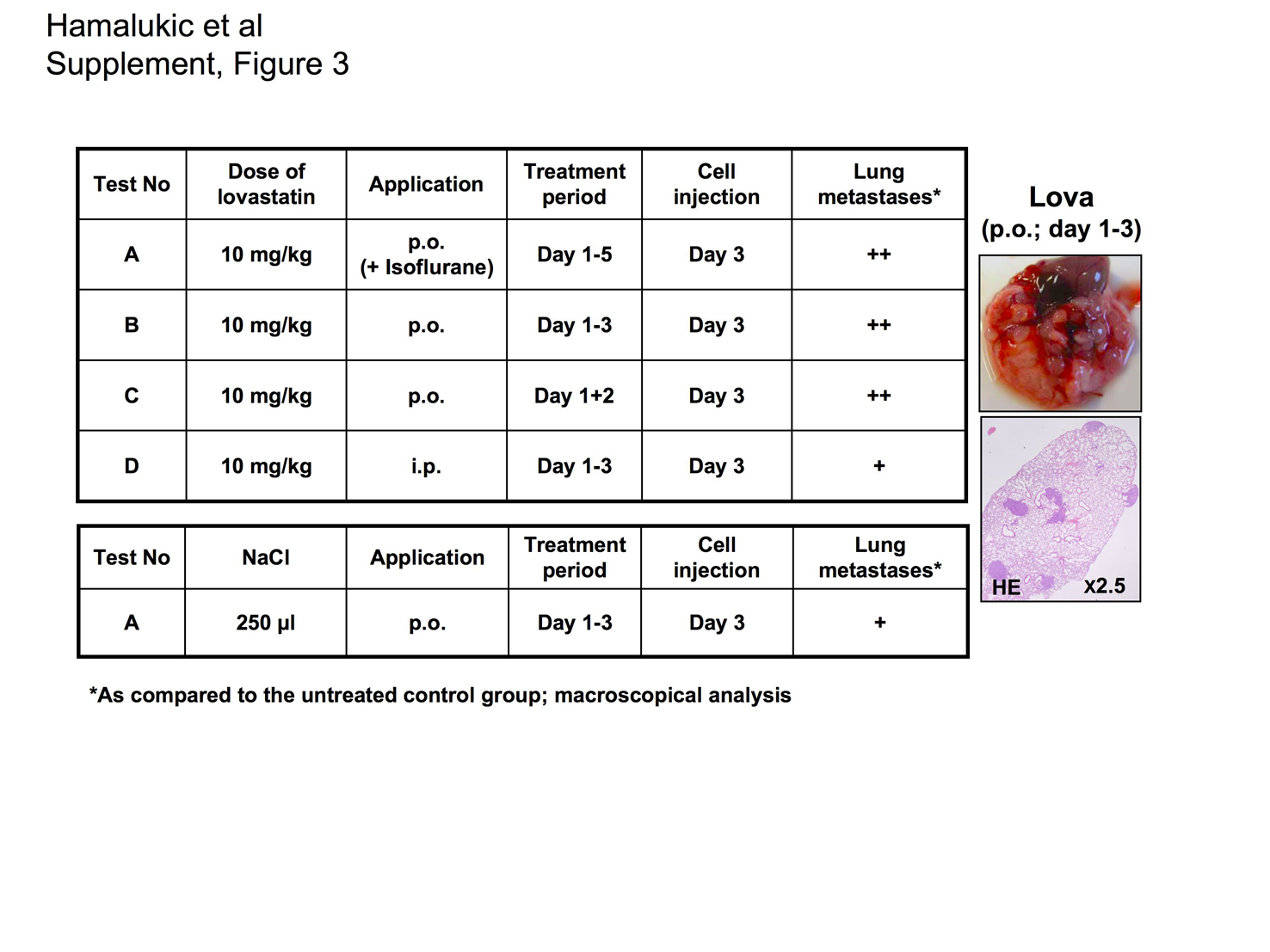

Supplement: Figure S3 — Effect of lovastatin on the formation of lung metastases. 2×10^6^ CHO-K1 cells were injected into the tail vein of Rag2−/− BALB/c mice which had been pretreated with lovastatin p.o. or i.p. for different periods of time. The formation of lung metastases was analyzed three weeks later. +, weak effect; ++, stronger effect. As further control, physiological NaCl solution was administered p.o. Data shown are based on the morphological analysis of n = 3–4 animals per experimental condition. (TIF) [file pone.0026413.s003.tif]

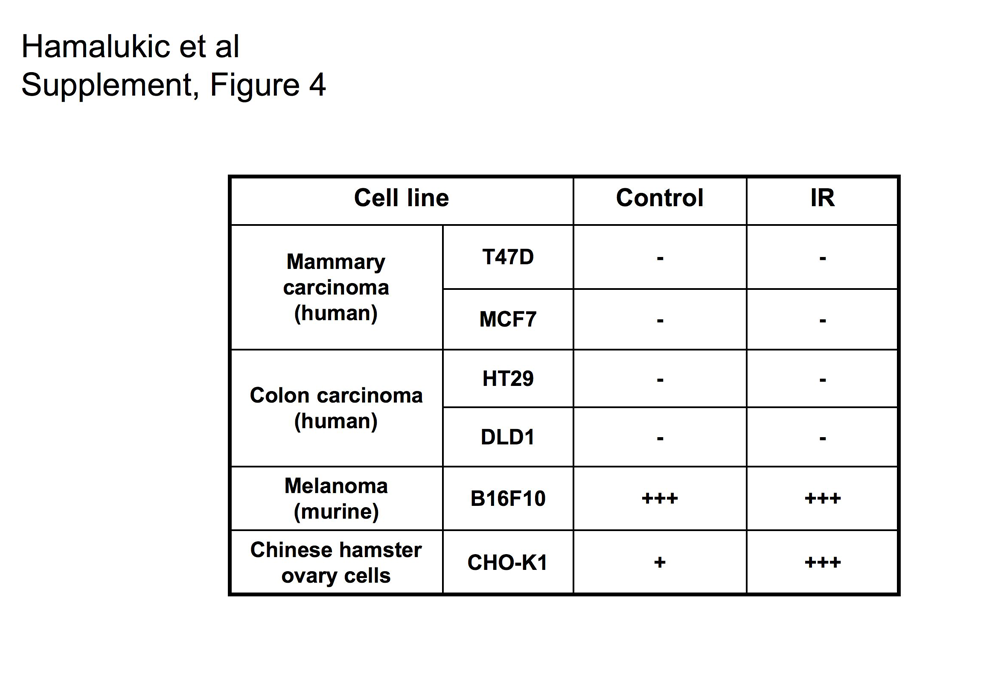

Supplement: Figure S4 — Cell line specificity of formation of lung metastases. 2×10^6^ cells were injected into the tail vein of Rag2−/− BALB/c mice. Afterwards mice were irradiated with 4 Gy (total body irradiation). The formation of lung metastases was analyzed three weeks later. Control, non-irradiated; IR, total body irradiation; -, no lung metastases detectable; +, weak effect; +++, strong effect. Data shown are based on the morphological analysis of n = 3–4 animals per cell line used. (TIF) [file pone.0026413.s004.tif]
